# Diagnostic Performance of 2D and 3D T2WI-Based Radiomics Features With Machine Learning Algorithms to Distinguish Solid Solitary Pulmonary Lesion

**DOI:** 10.3389/fonc.2021.683587

**Published:** 2021-11-18

**Authors:** Qi Wan, Jiaxuan Zhou, Xiaoying Xia, Jianfeng Hu, Peng Wang, Yu Peng, Tianjing Zhang, Jianqing Sun, Yang Song, Guang Yang, Xinchun Li

**Affiliations:** ^1^ Department of Radiology, The First Affiliated Hospital of Guangzhou Medical University, Guangzhou, China; ^2^ Philips Healthcare, Guangzhou, China; ^3^ Shanghai Key Laboratory of Magnetic Resonance, East China Normal University, Shanghai, China

**Keywords:** algorithms, area under the curve, lung neoplasms, machine learning, magnetic resonance imaging

## Abstract

**Objective:**

To evaluate the performance of 2D and 3D radiomics features with different machine learning approaches to classify SPLs based on magnetic resonance(MR) T2 weighted imaging (T2WI).

**Material and Methods:**

A total of 132 patients with pathologically confirmed SPLs were examined and randomly divided into training (n = 92) and test datasets (n = 40). A total of 1692 3D and 1231 2D radiomics features per patient were extracted. Both radiomics features and clinical data were evaluated. A total of 1260 classification models, comprising 3 normalization methods, 2 dimension reduction algorithms, 3 feature selection methods, and 10 classifiers with 7 different feature numbers (confined to 3–9), were compared. The ten-fold cross-validation on the training dataset was applied to choose the candidate final model. The area under the receiver operating characteristic curve (AUC), precision-recall plot, and Matthews Correlation Coefficient were used to evaluate the performance of machine learning approaches.

**Results:**

The 3D features were significantly superior to 2D features, showing much more machine learning combinations with AUC greater than 0.7 in both validation and test groups (129 vs. 11). The feature selection method Analysis of Variance(ANOVA), Recursive Feature Elimination(RFE) and the classifier Logistic Regression(LR), Linear Discriminant Analysis(LDA), Support Vector Machine(SVM), Gaussian Process(GP) had relatively better performance. The best performance of 3D radiomics features in the test dataset (AUC = 0.824, AUC-PR = 0.927, MCC = 0.514) was higher than that of 2D features (AUC = 0.740, AUC-PR = 0.846, MCC = 0.404). The joint 3D and 2D features (AUC=0.813, AUC-PR = 0.926, MCC = 0.563) showed similar results as 3D features. Incorporating clinical features with 3D and 2D radiomics features slightly improved the AUC to 0.836 (AUC-PR = 0.918, MCC = 0.620) and 0.780 (AUC-PR = 0.900, MCC = 0.574), respectively.

**Conclusions:**

After algorithm optimization, 2D feature-based radiomics models yield favorable results in differentiating malignant and benign SPLs, but 3D features are still preferred because of the availability of more machine learning algorithmic combinations with better performance. Feature selection methods ANOVA and RFE, and classifier LR, LDA, SVM and GP are more likely to demonstrate better diagnostic performance for 3D features in the current study.

## Introduction

A solitary pulmonary lesion (SPL) is one of the most common findings on chest radiographs and computed tomography (CT). An increasing number of pulmonary nodules are detected by CT with the improvement in lung cancer screening. However, most of these positive detections are not cancerous (1). The high false-positive rate can lead to a waste of medical resources, additional radiation exposure, unnecessary patient anxiety, and so on. Recent advances in magnetic resonance imaging (MRI) techniques make it possible to use lung MRI in routine clinical practice. Published evidence showed that lung MRI could be a potentially effective screening tool because its performance was comparable with that of low-dose CT (2), even with a lower false-positive rate for nodule detection (3). A conventional sequence, such as T2 weighted imaging(T2WI), has the potential to detect pulmonary nodules no less than 6 mm in diameter (4), which is essential for screening. However, as a morphological sequence, it may have limited value in distinguishing malignant from benign SPLs.

Radiomics is an emerging field that extracts a large number of quantitative features from medical imaging and quantifies tumor heterogeneity related to cellularity, necrosis, and angiogenesis in the tumor microenvironment (5). Therefore, radiomics provides the possibility for early and accurate diagnosis of SPLs. Radiomics can increase the diagnostic accuracy of baseline CT (6). In addition, studies have shown the potential of radiomics based on CT and MRI in distinguishing pulmonary lesions (7, 8). However, MR radiomics research focusing on differentiating SPLs has not yet been reported. Also, the performance of 2D and 3D CT features in pulmonary lesions has been shown to be controversial in different studies (9, 10). However, the performance of 2D and 3D MR features as well as their corresponding optimal machine learning methods in distinguishing SPLs has not been discussed. These issues are crucial for further generalization of MR radiomics in clinical research and application in the lung.

The present study aimed to develop and validate a T2WI-based radiomics classifier to differentiate between malignant and benign SPLs. In addition, different machine learning methods were evaluated to achieve the best performance. Furthermore, 2D and 3D features and their combination with clinical features were compared.

## Materials and Methods

### Data Cohort

This retrospective study was approved by the local ethics committee of the hospital, which waived the need for patients’ informed consent. Preoperative MRI data of 231 patients with chest lesions from November 2015 to April 2018 were analyzed. The inclusion criteria were as follows: (a) lesions were measurable on previous CT scan or T2-weighted imaging; (b) no contraindication for MR examinations; and (c) patients received no therapies or anti-inflammatory therapies at least 2 weeks before the MRI scan, and lesions showed no shrinkage. The exclusion criteria were as follows: (a) operations or biopsies were not available (*n* = 27); (b) multiple lesions were reported (*n* = 42); and (c) mediastinal or pleural neoplasms were found (*n* = 30).

Finally, 132 patients (men and women; age range, 19–78 years; mean age, 54.9 years) were included in the study. The histopathological examination revealed 93 malignant lesions (46 adenocarcinoma, 23 squamous carcinoma, 13 small cell carcinoma, 6 lymphoepithelioid carcinoma, 3 mucoepidermoid carcinoma, 1 sarcomatoid carcinoma, and 1 pulmonary synoviosarcoma). Benign lesions included 7 organizing pneumonia, 6 tuberculosis, 6 granulomatous inflammation, 6 pulmonary cryptococcoses, 4 pulmonary hamartomas, 3 pulmonary aspergilloses, 2 sclerosing alveolar cell tumors, 2 bronchial cysts with infection, 2 focal inflammation, and 1 pulmonary adenofibroma. The patients were randomly divided into two independent groups in a ratio of 7:3. The training cohort included 92 patients (65/27 = positive/negative), whereas the independent test cohort included 40 patients (28/12 = positive/negative).

### Image Data Acquisition

All patients were examined with a 3.0-T MRI (Achieva, Philips Healthcare, Best, The Netherlands) using a body phased-array coil. Turbo spin-echo T2-weighted (T2WI) imaging was performed using the following parameters: TR, 992 ms; TE 80 ms; field of view, 350 × 430 mm^2^; matrix, 640 × 640; thickness, 5 mm; gap, 0.5 mm.

### Lesion Segmentation

**Figure 1:**
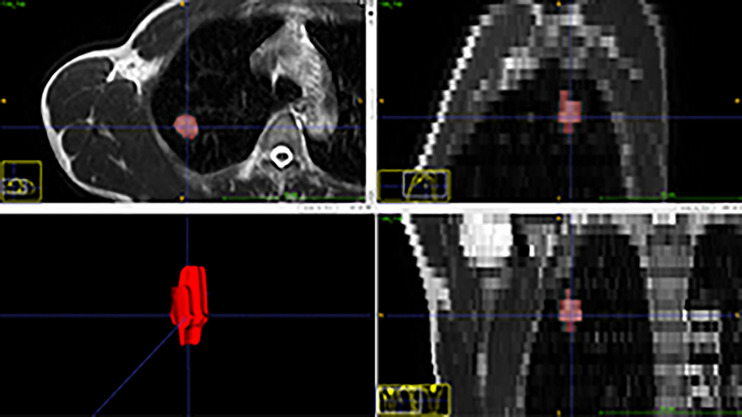
Segmentation of a nodule in the upper right lobe on T2-weighted images.

Mass segmentation was performed to select the entire tumor using open-source software (ITK-SNAP v. 3.6.0, http://www.itksnap.org). Regions of interest (ROIs) of lesions were segmented manually by the consensus of two radiologists with 3 and 8 years of experience (**Figure 1**). The ROIs included the whole tumor and excluded visible air regions.

### Extraction of Features

**Figure 2:**
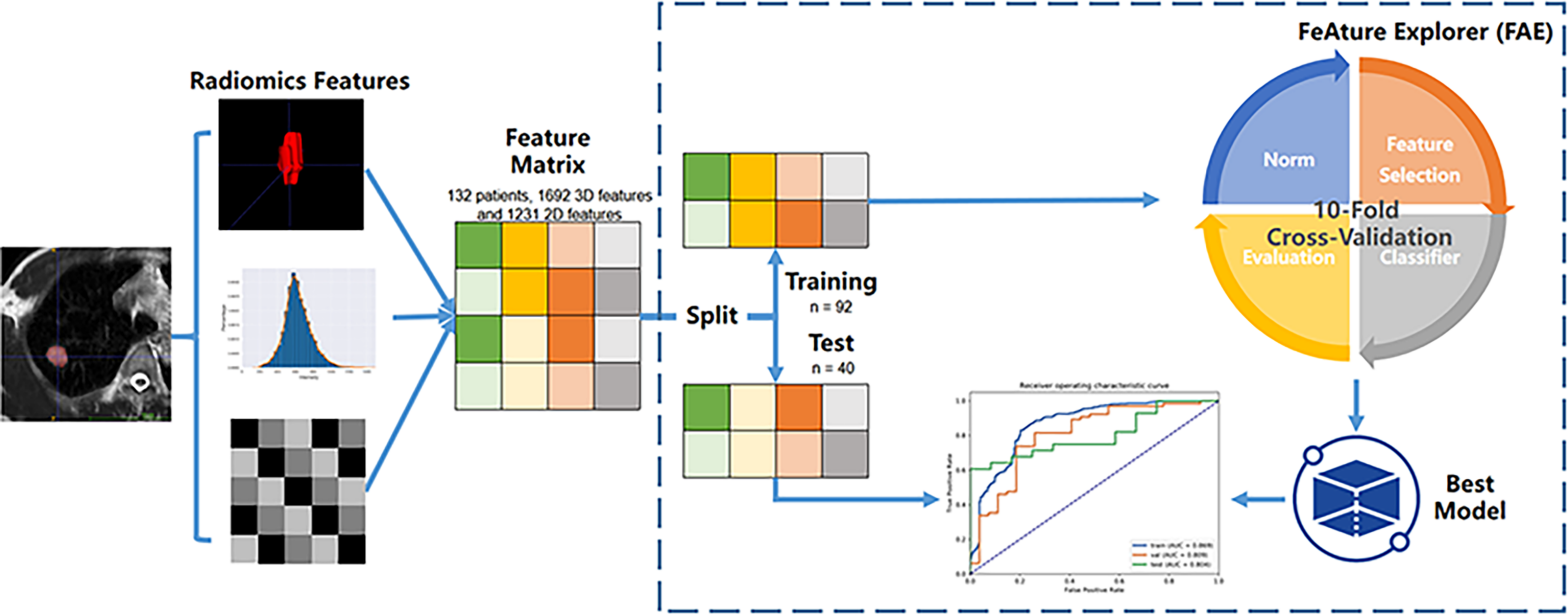
Flow chart for the data processing.

The radiomics features were extracted by the Philips Radiomics tool (Philips Medical Systems, Shanghai, China) based on pyRadiomics (11). The hyper-parameters were set to default parameters of the PyRadiomic. The details were described on the website: https://pyradiomics.readthedocs.io/en/latest/features.html. For each ROI, a total of 1,692 3D and 1231 2D radiomic features, including direct features, indirect features, Wavelet transform features, and Laplacian of Gaussian filtered features, were extracted as described in a previous study (12). The 2D features were generated using the slice with the maximum area in 3D ROI. The basic clinical data, sex and age, were included as clinical features. The flow chart for the data processing is displayed in **Figure 2**.

### Radiomics Feature Selection and Classifier Building

Building one machine learning model usually consisted of the following steps (1): normalizing each feature to avoid the effect of the scale (2); reducing the dimension of the feature space to remove the information of no use (3); selecting features from the remained features according to the label; and (4) training a classifier to map the selected features onto the diagnosis. In each step, different methods could be selected to make a machine learning pipeline for the final diagnosis.

**Figure 3:**
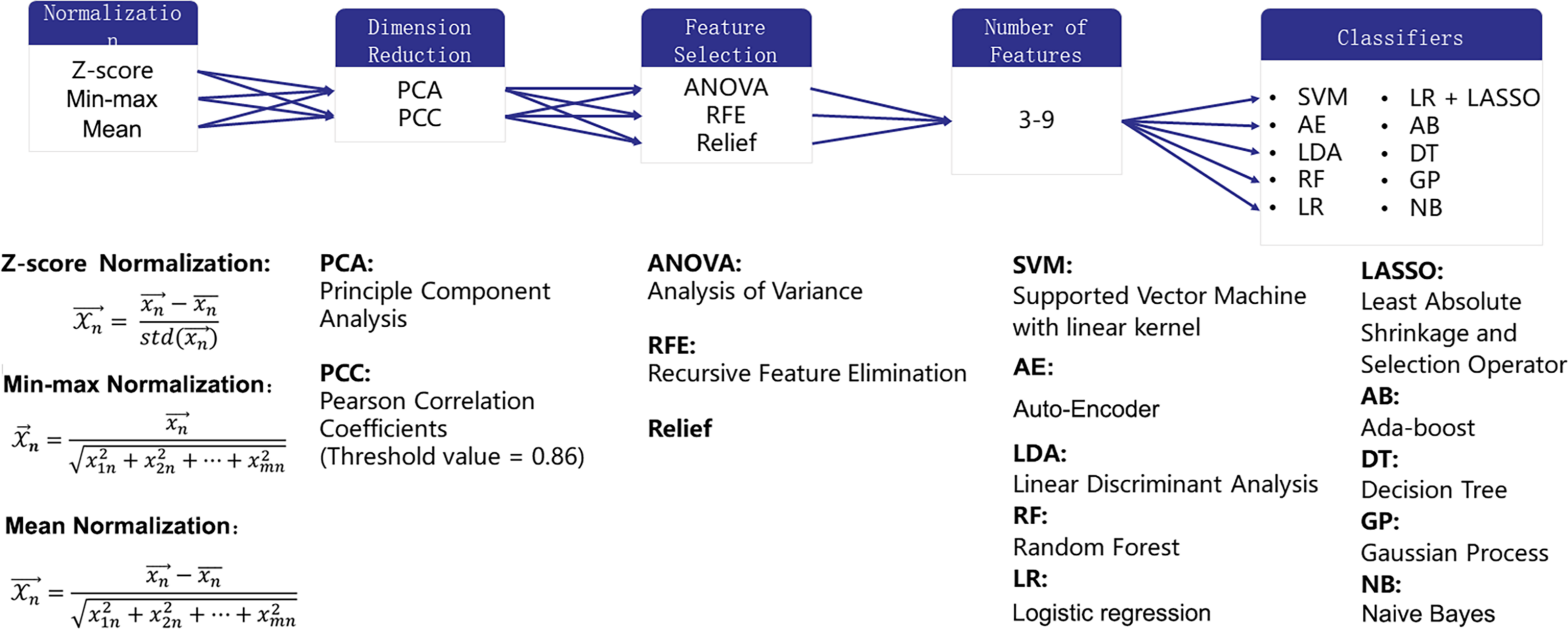
Combination of the pipelines for radiomics analysis.

Three normalization methods [Min-max Normalization (norm-unit), Z-Score Normalization (norm0center), Mean Normalization (norm0centerunit)], two dimension reduction algorithms [principal component analysis (PCA) and Pearson correlation coefficient (PCC)], three feature selection methods [analysis of variance (ANOVA), relief, recursive feature elimination (RFE)], and 10 different classifiers [support vector machine (SVM), auto-encoder (AE), linear discriminant analysis (LDA), random forest (RF), logistic regression (LR), LR-least absolute shrinkage and selection operator (Lasso), Adaboost (AB), decision tree (DT), Gaussian process (GP), naive Bayes (NB)] with 7 different feature numbers (confined to 3–9) were used to build 1260 models for the diagnosis. These methods were chosen due to their popularity in the literature. More details of the combination of the pipelines are illustrated in **Figure 3**. The number of features was constrained to less than 10% of the training sample size to build a robust machine learning model (13). The ten-fold cross-validation on the training dataset was applied to choose the candidate machine-learning pipeline. The pipeline was selected as the optimal final model when the area under the receiver operating characteristic (ROC) curve (AUC) difference between the training and validation sets on cross-validation was less than 0.1, and the AUC in the test set was the highest. Grid search is used for hyperparameter tuning. The search grid of each classifier was documented in **Supplementary Table 1**.

### Statistical Analysis

The clinical characteristics between the training and testing sets were compared using the Student *t* test for continuous variables and the chi-squared (*χ* ^2^) test for categorical variables. If the counting variable had a theoretical number <10, it was obtained by Fisher’s exact probability test. The aforementioned processes were executed in R software version 3.0.2 (The R Project for Statistical Computing, Vienna, Austria; http://www.r-project.org). A *P* value of <0.05 indicated statistical significance.

The performance of models was evaluated using the ROC curve, precision-recall(PR) plot, and Matthews Correlation Coefficient. The AUC-ROC and AUC-PR were calculated for quantification. The accuracy, sensitivity, specificity, positive predictive value (PPV), and negative predictive value (NPV) were also calculated at a cutoff value that maximized the value of the Youden index. Also, the estimation was boosted 1000 times to give the 95% confidence intervals (CIs) of AUC-ROC. The aforementioned processes were implemented with FeAture Explorer (14) (FAE, v0.2.5, https://github.com/salan668/FAE) on Python (3.6.8, https://www.python.org/), which is an open-source software based on scikit-learn (v0.22) (15).

## Results

### Clinical Characteristics

In general, a statistically significant difference was found in age (57.54 ± 10.24 vs. 49.23 ± 14.90, P < 0.001) and sex (male/female ratio 69:24 vs. 20:19, *P* = 0.01) between the malignant and benign groups. The clinical features of training and test cohorts are summarized in **Table 1**. No significant differences in lesion diameter were found between the training and test sets. Malignant tumors were more common in upper lobes, men, and elderly people in the training set (*P* = 0.039, 0.04, and 0.001, respectively) and showed a similar trend in the test set in an insignificant manner (*P* = 0.667, 0.121, and 0.13, respectively).

**Table 1:** Clinical features of training and test cohorts.

LABEL	Training cohort			Test cohort		
	benign	malignant	P-value	benign	malignant	P-value
**N**	27	65		12	28	
**Age**	48.22 ± 15.05	57.57 ± 10.71	0.001	51.50 ± 14.98	57.50 ± 9.26	0.13
**Diameter** **(cm)**	3.59 ± 2.54	4.51 ± 2.60	0.126	3.42 ± 2.28	4.60 ± 2.21	0.131
**Gender**			0.04			0.121
Male	15 (55.56%)	50 (76.92%)		5 (41.67%)	19 (67.86%)	
Female	12 (44.44%)	15 (23.08%)		7 (58.33%)	9 (32.14%)	
**Location**			0.039			0.677
Other lobes	20 (74.07%)	33 (50.77%)		6 (50.00%)	12 (42.86%)	
Upper lobe	7 (25.93%)	32 (49.23%)		6 (50.00%)	16 (57.14%)	

### Comparison of Different Machine Learning Classification Models

**Figure 4:**
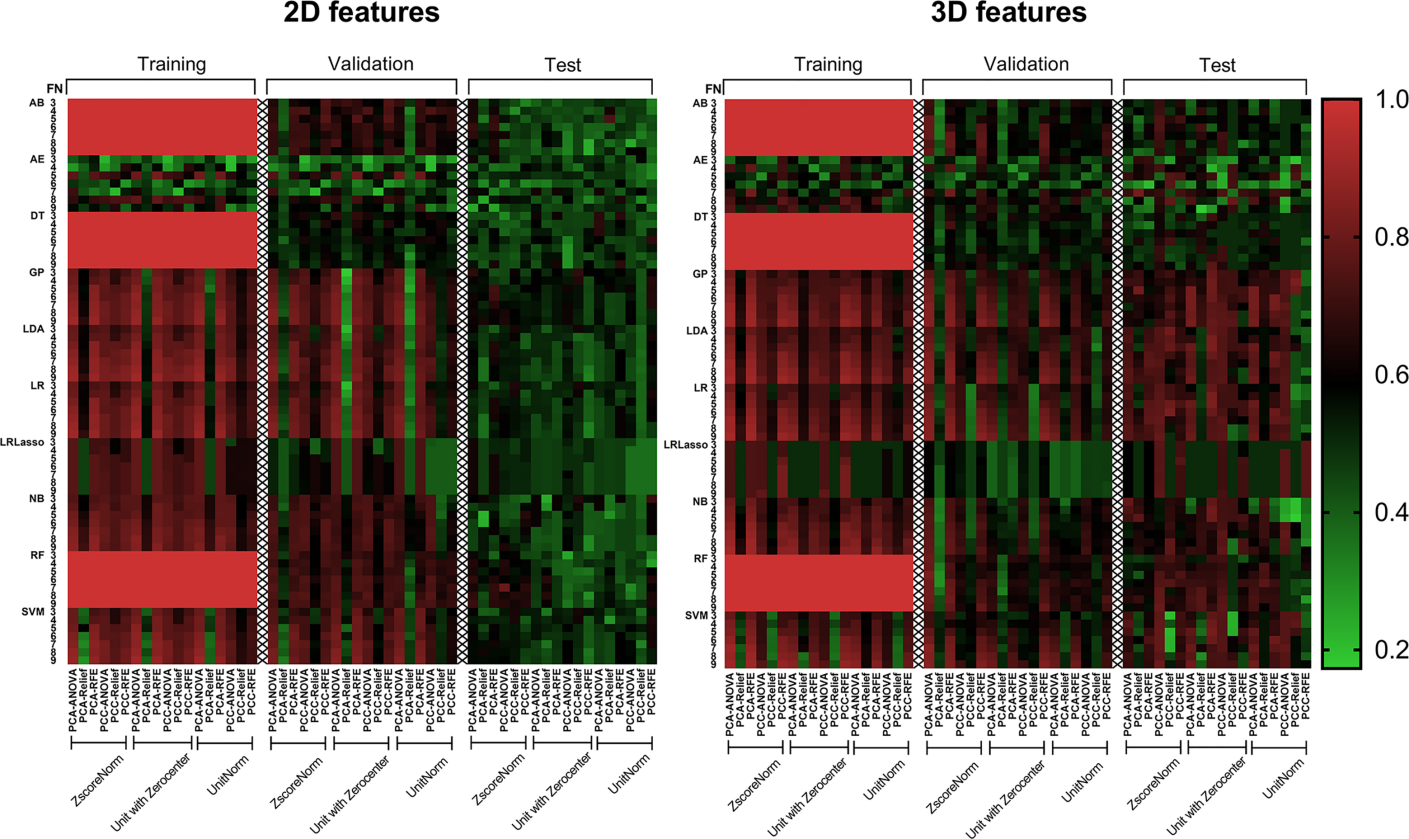
AUC heat map in each dataset showed the performance of 2D and 3D features combined with different machine learning methods in distinguishing solitary pulmonary lesions. It can be clearly seen that the 3D feature group has much more machine learning combinations with higher AUC than 2D feature group in the test dataset. AB, Adaboost; AE, auto-encoder; ANOVA, analysis of variance; DT, decision tree; FN, feature numbers; GP, Gaussian process; LASSO, least absolute shrinkage and selection operator; LDA, linear discriminant analysis; LR, logistic regression; NB, naive Bayes; PCA, principal component analysis; PCC, Pearson correlation coefficient; RF, random forest; RFE, recursive feature elimination; SVM, support vector machine; Unitnorm, Min-max Normalization; Unit with Zerocenter, Mean Normalization; Zscorenorm, *Z*-score normalization.

**Figure 4** shows the AUC heat map of 2D and 3D features with different machine learning methods. The number of models with AUC greater than 0.7 in both the validation and test groups was used as an evaluation index. A total of 140 models based on 3D features (*n* = 129) and 2D features (*n* = 11) showed AUC greater than 0.7 in both groups. For dimension reduction algorithms, PCA (*n* = 111) showed higher performance than PCC (*n* = 29). In terms of normalization, min-max, Z-score, and mean normalization had similar performance in 3D features, while models using only the Z-score (*n* = 11) showed AUC >0.7 for 2D features. For feature selection, ANOVA (*n* = 80) performed the best followed by RFE (*n* = 60), while relief had poor performance (*n* = 0) in the dataset. As for classifiers, SVM, LDA, LR, GP, and NB performed better for 3D features, while RF, AB, and GP performed better for 2D features (**Table 2**).

**Table 2:** The number of models with AUC greater than 0.7 in both validation and test groups.

		AUC_val_ > 0.7 &AUC_test_ > 0.7		AUC_val_> 0.7 &AUC_test_ > 0.8	
		3D features	2D features	3D features	2D features
**Dimension reduction**	PCA	101	10	22	0
	PCC	28	1	1	0
**Normalization**	Min-max	36	0	13	0
	Z-score	45	11	1	0
	Mean	48	0	9	0
**Feature selection**	ANOVA	75	5	17	0
	RFE	54	6	6	0
	Relief	0	0	0	0
**Classifier**	SVM	24	0	5	0
	AE	0	0	0	0
	LDA	33	0	3	0
	RF	0	5	0	0
	LR	41	0	3	0
	LR + LASSO	0	0	0	0
	AB	0	4	0	0
	DT	0	0	0	0
	GP	23	2	12	0
	NB	8	0	0	0

AUC, area under the curve; PCA, principal component analysis; PCC, Pearson Correlation Coefficients; ANOVA, analysis of variance; RFE, recursive feature elimination; SVM, support vector machine; AE, auto-encoder; LDA, linear discriminant analysis; RF, Random forest; LR, logistic regression; LASSO, least absolute shrinkage and selection operator; AB, Adaboost; DT, Decision Tree; GP, Gaussian Process; NB, Naive Bayes.

### Model Performance

**Figure 5:**
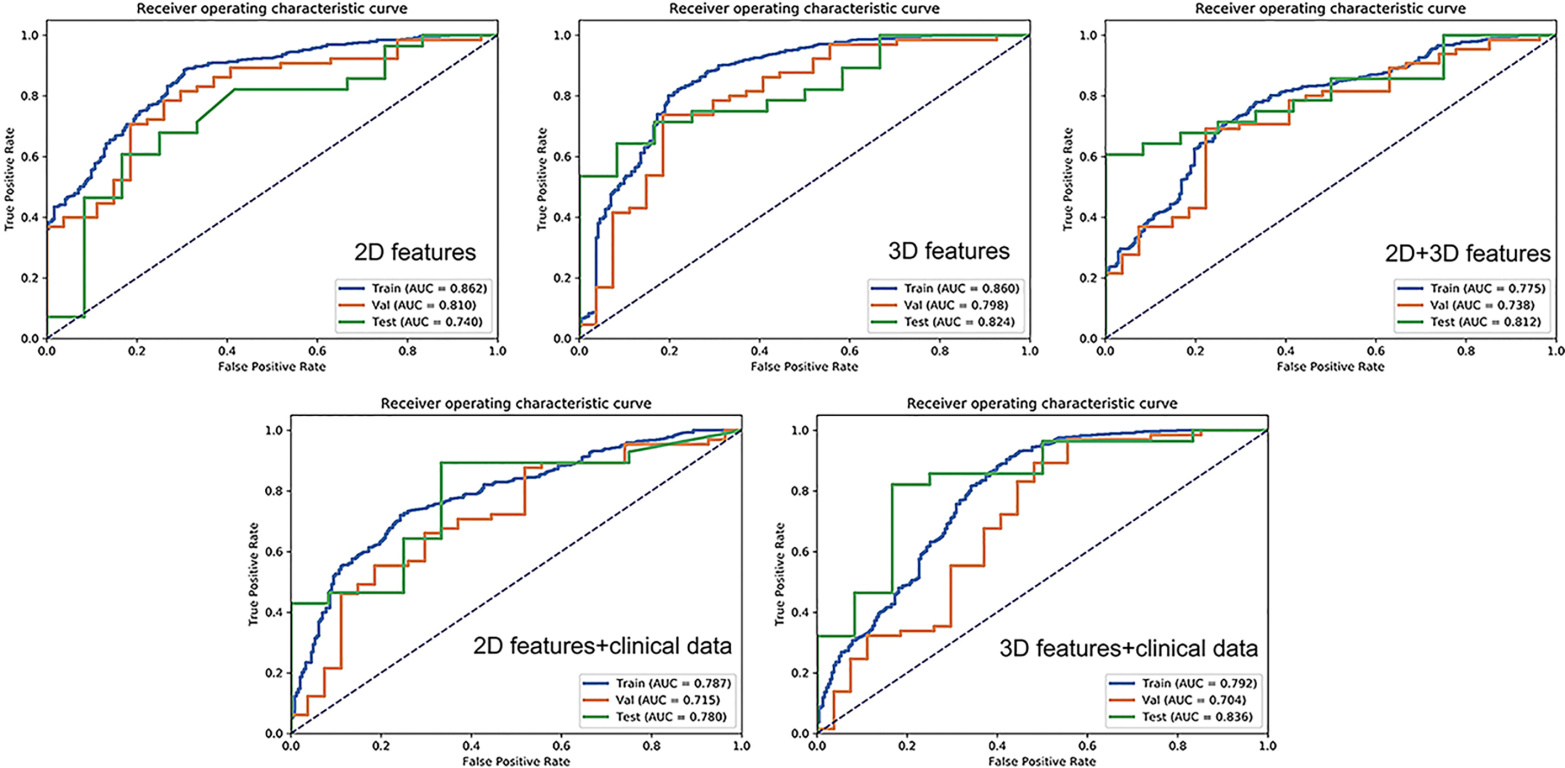
Receiver operating characteristic curves for 2D features, 2D + clinical features, 2D + 3D features, 3D features, and 3D + clinical features in distinguishing malignant from benign solitary pulmonary lesions.

**Figure 6:**
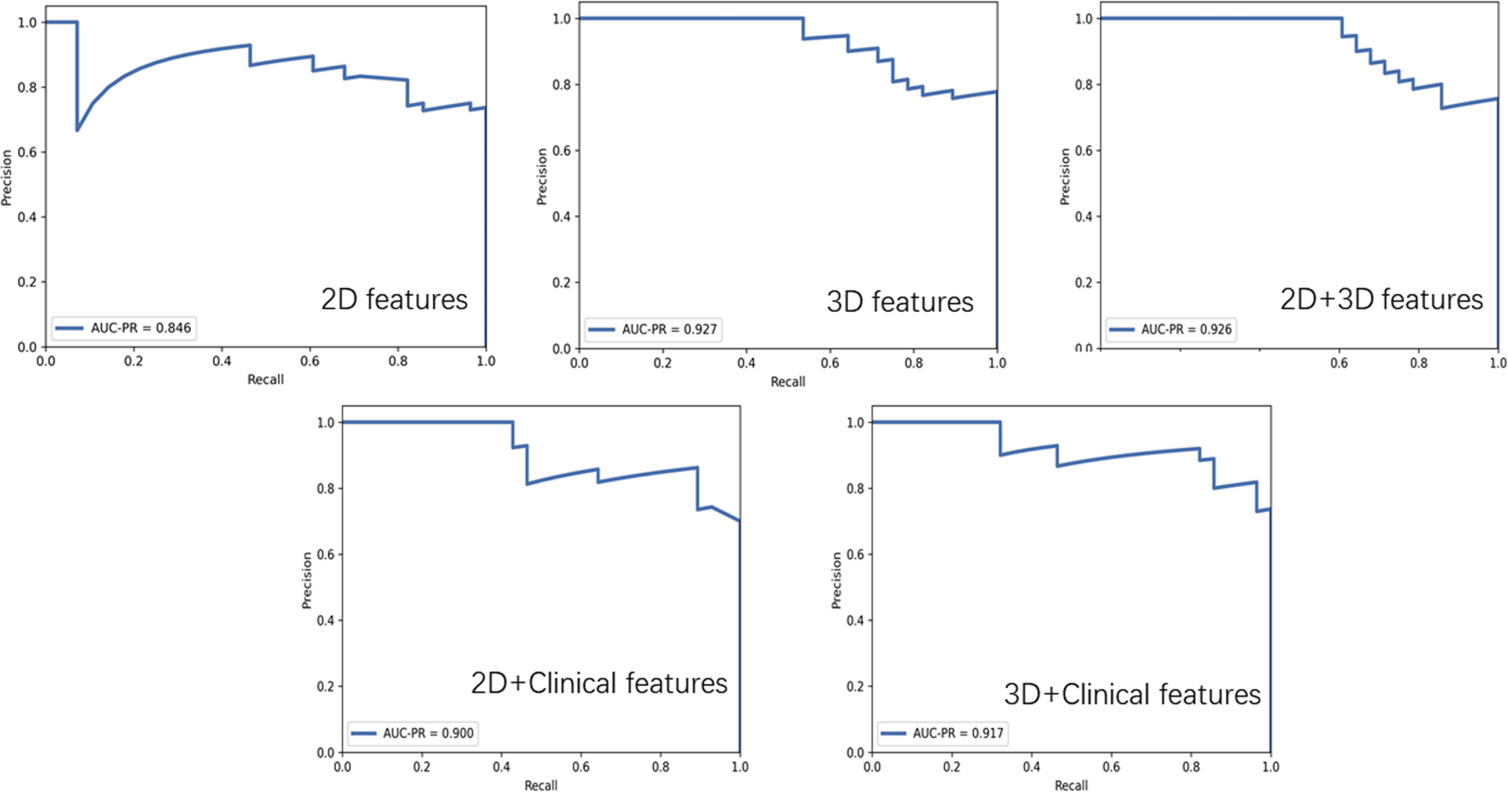
The precision-recall plots of optimal models based on different features.

The AUC-ROC and AUC-PR of different models are shown in **Figures 5**, **6**. For 2D features, the model based on six features with Z-score + PCA + RFE + GP could achieve stable (the difference in the AUC of the training and validation sets was less than 0.1) and the highest AUC in the test dataset (training: 0.858; validation: 0.810; test: 0.740) with a sensitivity of 0.607 and specificity of 0.833. The model obtained an AUC-PR of 0.846 and MCC of 0.404. After adding clinical features, the AUC-ROC, AUC-PR, and MCC in the test dataset were improved to 0.780, 0.900, and 0.574 (sensitivity: 0.893, specificity: 0.667) with a machine learning pipeline of Z-score + PCA + ANOVA + SVM.

For 3D features, the model based on nine features with Min-max + PCA + ANOVA + GP could achieve stable and the highest AUC (training: 0.858; validation: 0.797; test: 0.824) with a sensitivity of 0.643 and specificity of 0.917. The model obtained an AUC-PR of 0.927 and MCC of 0.514. Incorporating clinical features with 3D radiomic features slightly improved the AUC-ROC of the testing dataset (AUC = 0.836, sensitivity: 0.821, specificity: 0.833), with an AUC-PR of 0.918 and MCC of 0.620.

For the joint 2D and 3D features, the model based on nine features with Z-score + PCC + ANOVA + SVM could achieve stable and the highest AUC (training: 0.770; validation: 0.737; test: 0.813) with a sensitivity of 0.607 and specificity of 1.000. The model obtained an AUC-PR of 0.926 and MCC of 0.563. The clinical statistics of the independent test dataset are summarized in **Table 3**. The hyperparameter settings of each classifier used in final models were provided in **Supplementary Table 2**.

**Table 3:** Clinical statistics in the independent test dataset.

	AUC-ROC	95% CI	AUC-PR	MCC	Sen	Spe	PPV	NPV	P-value
2D features	0.740	0.716-0.800	0.846	0.404	0.607	0.833	0.895	0.476	<0.001
2D features + Cli	0.780	0.763-0.81	0.900	0.574	0.893	0.667	0.862	0.727	<0.001
3D features	0.824	0.808-0.851	0.927	0.514	0.643	0.917	0.947	0.524	<0.001
3D features + Cli	0.836	0.821-0.883	0.918	0.620	0.821	0.833	0.920	0.667	<0.001
Joint 2D&3D features	0.813	0.796-0.833	0.926	0.563	0.607	1.000	1.000	0.522	<0.001

AUC, area under the curve; ROC, receiver operator characteristic curve; Cli, clinical features; CI, confidence interval; PR, precision-recall plot; MCC, Matthews Correlation Coefficient; Sen, sensitivity; Spe, specificity; PPV, positive predictive value; NPV, negative predictive value.

## Discussion

The identification of optimal machine learning methods is essential for stable and clinical application (16). The present study provided a comprehensive and detailed assessment of machine learning approaches and explored the diagnostic value of multiple models including 2D, 3D radiomics models, and combinations of clinical and radiomics models based on MR T2WI in noninvasively differentiate SPLs. Our results demonstrated that the T2WI-based radiomics model showed potential in differentiating malignancy from benign SPLs. The 3D radiomics features were better than the 2D features in differentiating SPLs. The optimal machine learning methods were not consistent in different scenarios or with different features included.

Both 2D and 3D features have been employed in previous radiomics researches. A previous CT study suggested that 2D features were superior to 3D features in predicting the prognosis of non-small cell lung cancer (10). However, in some other studies, 3D features demonstrated better predictive performance (17, 18). In the present study, the number of extracted 2D radiomics features was less than that of 3D features because 2D features were extracted based on a single slice, thus losing the spatial information within lesions. Therefore, some features reflecting the spatial distribution of voxels became unavailable. The study found that the radiomics signature derived from 3D features outperformed the signature from 2D features, indicating that the 3D volumetric ROI contained more comprehensive information than 2D ROI and therefore had a better diagnostic performance. Although joint 2D and 3D features showed a higher AUC in a previous study (9), they failed to show superiority over 3D features in the present study. Instead, the performance of joint features was similar to that of 3D features. This finding suggested that the joint features contained information on both 2D and 3D features, while 2D features could not provide new information to 3D features in the present cohort. Accordingly, the classification performance of the joint model failed to show improvement.

In this study, the dimensionality reduction method of PCA was better than PCC, probably because each feature was linearly independent after PCA. Therefore, information could be expressed with fewer features, and thus the performance was stable. However, PCA made variables less interpretable. Therefore, PCA was not suitable for cases where feature “interpretability” was emphasized. In addition, In this study, the three normalization methods (min-max, Z-score, and mean normalization) showed little difference in 3D features, indicating that all these three can be used as effective normalization methods.

Our results showed that the feature selection methods ANOVA and RFE, and the classifier LR, LDA, SVM and GP yield relatively better diagnostic performance for 3D features compared with other methods. This explains the optimal machine learning approach reported in some previous studies (14, 19). Song et al. (14) reported that ANOVA feature selection and an LDA classifier yielded the highest AUC in classifying the clinical-significant prostate cancer (CS PCa) and non-CS Pca. Wang et al. (19) reported that RFE combined with SVM performed the best in distinguishing benign and malignant pulmonary lesions. These also suggested that the optimal machine learning methods were not consistent in various scenarios. Besides, we found that the optimal machine learning strategy was nonunique and different among 2D, 3D, and joint features, indicating that the optimal method might vary depending on the features included.

The lesion diameter did not differ between benign and malignant groups. This is due to the inclusion of inflammatory lesions in the benign group, which could be patchy and thus have a large diameter. Besides, the results showed that elderly patients and men were predisposed to lung malignancies, which was consistent with the findings of a previous study (20). Integrating these clinical data into the radiomics model further increased the accuracy of the model, indicating that clinical and radiomics features contained complementary information needed for differential diagnosis. However, the improvement after adding clinical data was not significant in the present cohort. This might be because the difference in sex and age between malignant and benign groups was not significant in the test dataset. The radiomics models (especially with 3D features) still performed well under such circumstances, which suggested that the radiomics models had the potential to differentiate SPLs in patients with an atypical clinical history (e.g., lung cancer in young patients).

The present study found that some models had pretty high AUC in the training group but relatively lower AUC in the validation or test group (e.g., the classifier RF and AB achieved AUC of 1.0 in the training group). This could be attributed to overfitting and should be avoided. This finding confirmed that it was necessary to have independent datasets to test the real performance of the developed radiomics model. Based on the experience, it was preferred that the difference in AUC between the training and validation or test groups was less than 0.1, indicating that the model was more stable. In the present study, the final models had good discrimination efficiency in the training cohort and showed similar performance in the validation and test groups, suggesting that the models developed in this study were robust.

Previous studies conducted on CT demonstrated that the radiomics model was helpful in distinguishing pulmonary lesions (8, 21–23). Chen et al. (23) found that the accuracy of the radiomics signature in benign or malignant classification was 84% with a sensitivity of 92.85% and a specificity of 72.73%. Choi et al. (22) developed a radiomics model with an accuracy of 84.6%, which was 12.4% higher than that of lung-RADS. The performance of the proposed model in the present study was relatively satisfactory and promising, which was similar to that reported in CT radiomics studies. MRI showed great potential in lung application with the advantage of radiation-free and multi-parametric imaging. Recently, pulmonary nodule characterization using MR is recommended for clinical use (24). Nonetheless, the application of MR radiomics in assessing lung diseases is in the initial stage. Therefore, the potential clinical significance of our research is that, for one thing, it provides a non-invasive method that may help to increase the accuracy of MR routine sequence in the differentiation of SPL, for another, it lays a theoretical basis for more clinical applications of MR radiomics in the lung in the future.

This study had several limitations. First, the retrospective study design was subject to potential selection bias. Second, PCC threshold 0.9 has been used in previous studies to screen radiomics features (25, 26). In this study, we used a threshold value lower than 0.9 to more rigorously filter out redundant features. However, a seemingly arbitrarily chosen value of 0.86 was used since in the software we used, PCC can only be set as 0.86 by default. Third, the study included only T2WI to reduce the impact of parametric changes because its scanning parameter remained consistent in all patients while other sequences did not. However, other sequences such as T1W contrast enhancement, diffusion-weighted imaging, and ultrashort TE MRI (27) could provide valuable information and should be included in future studies. Fourth, lesion segmentation was not automatic in the present study and thus could be susceptible to potential human error. Finally, the sample size was relatively small. Therefore, a study with larger sample size and external validation from another institute is needed.

In conclusion, the present study developed and validated a radiomics model based on T2WI that might serve as a promising tool for noninvasive discrimination of SPLs. The 3D features were better than 2D features in differentiating SPLs and performed well in populations with different clinical characteristics. Therefore, 3D segmentations are recommended for further MR radiomics researches. Combining radiomics features with clinical data could further improve model performance. Nonetheless, the optimal machine learning method might not be consistent in different scenarios or with different features involved.

## Data Availability Statement

The original contributions presented in the study are included in the article. Further inquiries can be directed to the corresponding author.

## Ethics Statement

The studies involving human participants were reviewed and approved by the First Affiliated Hospital of Guangzhou Medical University. Written informed consent for participation was not required for this study in accordance with the national legislation and the institutional requirements. Written informed consent was not obtained from the individual(s) for the publication of any potentially identifiable images or data included in this article.

## Author Contributions

QW and XL conceived the project. QW and JZ completed the article writing. XX, JH, PW, and YP completed data collection and statistical analysis. QW, TZ, and JS completed the Radiomics analysis and chart making. YS and GY provided technique support. All authors contributed to the article and approved the submitted version.

## Funding

This study is supported by National Natural Science Foundation of China  (81601457) the Foundation of Guangzhou Municipal Science and Technology Bureau (202102010253), and Open Project Fund of the Sixth Affiliated Hospital of Guangzhou Medical University (2020–11–370).

## Conflict of Interest

Authors TZ and JS were employed by Philips Healthcare.

The remaining authors declare that the research was conducted in the absence of any commercial or financial relationships that could be construed as a potential conflict of interest.

## Publisher’s Note

All claims expressed in this article are solely those of the authors and do not necessarily represent those of their affiliated organizations, or those of the publisher, the editors and the reviewers. Any product that may be evaluated in this article, or claim that may be made by its manufacturer, is not guaranteed or endorsed by the publisher.
