# Supplementary material for: Diagnostic Performance of 2D and 3D T2WI-Based Radiomics Features With Machine Learning Algorithms to Distinguish Solid Solitary Pulmonary Lesion
Source: Front Oncol. 2021 Nov 18;11:683587. doi: 10.3389/fonc.2021.683587 (PMC8637439; doi:10.3389/fonc.2021.683587)
Supplement: Supplementary file 3 [file Table_1.docx]

Supplementary Table 1. The grid search parameter settings of each classifier

| Classifiers | Grid search parameter settings |
| --- | --- |
| AdaBoost | We search the number of the estimators in [10, 30, 50, 80, 100] |
| Auto-Encoder | The size of hidden layer was in [30, 60, 100], the learning rate was in [0.01, 0.001], the weight of L2 constrain was in [0.001, 0.0001] |
| Decision Tree | The criterion was searched in ['gini', 'entropy'], and the number of maximum features was search in ['auto', 'sqrt', 'none'] |
| Linear Discriminant Analysis | The solver was set in ['svd', 'lsqr', 'eigen'] |
| Logistic Regression | The tol was wet in [0.0001, 0.001, 0.01]. C was set in [0.01, 0.1, 1, 10] |
| Random Forest | The number of estimators was in [10, 50, 100], the criterion was set in ["gini", "entropy"] |
| Supported Vector Machine | The kernel was set to 'linear' with a range of C in [0.1, 0.3, 1, 3]. The kernel was set to 'rbf' with a range of gamma in [0.1, 0.3, 1, 3]. The kernel was set to 'poly' with a range of degree of [2, 3, 4] |

Note: Hyperparameters of Gaussian process, Naive Bayes, and Least absolute shrinkage and selection operator used the default setting. Please refer to the full user guide of scikit-learn for further details (https://scikit-learn.org/stable/index.html).

Supplementary Table 2. The parameter settings of each classifier used in final models

|  | **The classifier used in the final model** | **Parameter settings** |
| --- | --- | --- |
| 2D features | Gaussian process | **Optimizer**=fmin_l_bfgs_b |
| 3D features | Gaussian process | **Optimizer**=fmin_l_bfgs_b |
| Joint 2D&3D features | Supported Vector Machine | **penalty**=l2, **loss**=squared_hinge, **C**=1.0 |
